# Nonlinear response characteristics of neural networks and single neurons undergoing optogenetic excitation

**DOI:** 10.1162/netn_a_00154

**Published:** 2020-09-01

**Authors:** Jannik Luboeinski, Tatjana Tchumatchenko

**Affiliations:** Theory of Neural Dynamics Group, Max Planck Institute for Brain Research, Frankfurt am Main, Germany; Department of Computational Neuroscience, III. Institute of Physics – Biophysics, University of Göttingen, Göttingen, Germany; Theory of Neural Dynamics Group, Max Planck Institute for Brain Research, Frankfurt am Main, Germany

**Keywords:** Optogenetics, Channelrhodopsin, Neural networks, Nonlinear response characteristics

## Abstract

Optogenetic stimulation has become the method of choice for investigating neural computation in populations of neurons. Optogenetic experiments often aim to elicit a network response by stimulating specific groups of neurons. However, this is complicated by the fact that optogenetic stimulation is nonlinear, more light does not always equal to more spikes, and neurons that are not directly but indirectly stimulated could have a major impact on how networks respond to optogenetic stimulation. To clarify how optogenetic excitation of some neurons alters the network dynamics, we studied the temporal and spatial response of individual neurons and recurrent neural networks. In individual neurons, we find that neurons show a monotonic, saturating rate response to increasing light intensity and a nonmonotonic rate response to increasing pulse frequency. At the network level, we find that Gaussian light beams elicit spatial firing rate responses that are substantially broader than the stimulus profile. In summary, our analysis and our network simulation code allow us to predict the outcome of an optogenetic experiment and to assess whether the observed effects can be attributed to direct or indirect stimulation of neurons.

## Author Summary

Optogenetic circuit manipulation has become a popular tool to manipulate the activity of neurons. During optogenetic stimulation, the firing rate of a neuron can rise because of direct light excitation or indirect activation via other neurons. To disentangle these influences and predict the effects of optogenetic excitation, we set up a spiking network model with controlled connectivity and studied its response to light stimulation. We find that the optogenetically evoked activity in a network can spread far beyond the light-stimulated area. We further found a nonmonotonic rate response of single neurons to increasing light intensities and frequencies. Our results help to interpret optogenetic experiments in vivo, and we provide computer code that can be customized to simulate 2D connectivity scenarios and explore their consequences.

## INTRODUCTION

Over the last several years, the field of optogenetics has led to the development of extremely useful experimental tools that can be employed to stimulate single cells or entire neuronal populations. Essentially, optogenetics enables precisely targeted stimulation of specific cell typesby genetic modification and exposure to light. Optogenetic techniques have already facilitated many explorations, and likely many more will follow (Deisseroth, 2015).

An experimentally well-studied class of optogenetic tools are channelrhodopsins (Berndt et al., 2011; Lin, 2011; Schneider, Grimm, & Hegemann, 2015). Being a light-gated ion channel that leads to excitation of its host cell, wild-type channelrhodopsin-2 (ChR2) was the first microbial rhodopsin successfully employed as an optogenetic tool (Boyden, Zhang, Bamberg, Nagel, & Deisseroth, 2005; Nagel et al., 2003). Nowadays, a multitude of engineered variants of ChR2 exist. The work we present here is based on a model of the ChR2/H134R variant, which is popular for its enhanced photocurrents as well as for having a good peak/steady-state ratio and increased light sensitivity (Lin, 2011; Tchumatchenko, Newman, Fong, & Potter, 2013; Williams et al., 2013; Yawo, Asano, Sakai, & Ishizuka, 2013). The ChR2/H134R variant, however, is only an example of an optogenetic channel that can be used with our model. Parameter modifications and customization of the simulational tools which we provide will facilitate analogous network studies for other optogenetic variants.

Making quantitative activity predictions for neuronal networks in vivo is complicated by the interaction of the different timescales of rhodopsins, neurons, and synapses, as well by the voltage dependence of conductances. Determining the stimulus-response relationship in a network by simultaneously measuring the activities of thousands of neurons is experimentally challenging, and only recently pioneering experiments (Chettih & Harvey, 2019; Daie, Svoboda, & Druckmann, 2019; Russell et al., 2019) started tackling this question. Here, we present theoretical results that aid in the prediction of experimental outcomes as experiments push in the direction of understanding how populations of neurons respond to specific stimuli (Humphries, 2017). Hence, a reliable prediction of the network effects evoked by optogenetic excitation of a defined cell population would be an extremely helpful tool. Theoretical studies on the network response to pulsed current stimulation are not sufficient to describe optogenetic stimulation because the resonance properties of the rhodopsins have a complex effect on the properties of single neurons and whole networks. By including a detailed channel model in a network setting, however, optogenetically evoked network responses can be predicted more accurately. Proposing such a model, we aim to bridge the gap between channel dynamics and spiking, in addition to providing predictions for network and single-neuron dynamics following optogenetic stimulation.

We constructed our computational model by using a network of Leaky Integrate-and-Fire neurons, which are biologically realistic and have been used in a number of studies addressing the response of cortical populations (Fourcaud-Trocmé, Hansel, van Vreeswijk, & Brunel, 2003; Gerstner & Naud, 2009; Tchumatchenko, Malyshev, Wolf, & Volgushev, 2011). We augmented the Leaky Integrate-and-Fire neuron model by including in its input a population of ChR2 channels, simulated with a three-state Markov model previously investigated by others (Nikolic et al., 2009; Tchumatchenko et al., 2013; Williams et al., 2013). Given its few parameters and short runtime, the three-state model is a prime candidate for large network simulations. In addition, the model’s good response match to continuously varying stimuli (Tchumatchenko et al., 2013) made us choose the three-state model over a competing four-state model (Nikolic et al., 2009; Williams et al., 2013).

In the channel model we consider, each individual ChR2 channel can only be in one of the three states at a time. For the thousands of channels that are typically found in the membrane of ChR2-transfected neurons, a stochastic description via three continuous probability variables is sufficient. The expression level of ChR2 (i.e., the number of channels in a neuron) is difficult to determine in experiments, so that only rough estimations of its magnitude can be made. Here, we study two expression levels: a low expression level (60,000 channels per neuron) and a high expression level (300,000 channels per neuron). We chose these values because we found through simulations that 60,000 channels per neuron by themselves, without external or recurrent input, are not sufficient to trigger spikes, while the stimulation of a neuron with 300,000 channels reliably correlates with spiking. We estimated these numbers from conductance values in experiments, using them as representative values for relatively low and high expression of ChR2 (Nagel et al., 2003; Zhang, Wang, Boyden, & Deisseroth, 2006). The lower expression level yielded a small, but measurable, correlation between light pulses and spikes, and can therefore be considered a subthreshold modulation in in vivo networks. The higher level evoked spikes that were nearly phase-locked to the light pulses.

To evoke network responses, we chose a periodic stimulation with a frequency of 50 Hz, which is commonly used in experiments (Lin, Lin, Steinbach, & Tsien, 2009; Zhang et al., 2006), and we investigated the response across a broad range of light intensities. To monitor the impact of the connection probability on the network response, we also varied the connection probability across a broad range of values. Furthermore, we aimed to obtain a detailed characterization of the single neuron response by considering a large number of frequencies and light intensities.

If the network response were linear, we could expect a constant spatial profile at all stimulation amplitudes. We found, however, that the spatial distribution of activities in realistic, nonlinear neural networks is much broader than the spatial light stimulation, and that the width of this activity distribution depends on the light intensity, as well as on the number of channels expressed in neurons. Thus, our main finding is that a narrow stimulation profile evokes a broad response profile. We further explored the nonlinear dependence of the peak and the baseline activity as well as the mean activity in the network as a function of the light intensity.

Interestingly, for single neurons under pulsed light stimulation we discovered that the magnitude of the firing rate response minimum increases with higher stimulation frequency, while the absolute height of the evoked response pulses decreases. Moreover, we found the duration of the firing rate response pulses to exhibit a nonmonotonic relation to the stimulation frequency. With respect to the light intensity, we found a monotonic saturation of the firing rate response.

Our results show that the stimulus-response relationship of networks and single neurons exhibiting ChR2 is highly nonlinear but can, nevertheless, be described in a tractable manner. We provide a characterization of this nonlinear behavior, along with our customizable simulation tools, which can be used to predict the ChR2-mediated response of networks and of single neurons to pulsatile stimulation protocols. Our model and our results contribute a quantitative perspective and offer tractable in silico predictions that can be used to design experiments.

## METHODS

### Software Implementation

For fitting, we used gnuplot (version 5.0.3) and GNU Scientific Library (version 2.4). We also used gnuplot to create plots. We implemented all of our simulations in C++ (ISO 2011 standard) and compiled them with g++ in version 7.5.0. The source code and ready-to-run binary versions of our simulation tools can be retrieved from https://github.com/jlubo/nn-lightchannels-sim or from https://jlubo.net/nn-lightchannels-sim.

### Channel and Neuron Model

Our model assumes a channel to be in one of the following three states:

1.  closed (with probability *C*): no current is flowing, activation by effective photon flux *ϵϕ*(*t*) is possible;
2.  open (with probability *O*): current is flowing, desensitization will occur stochastically at rate Γ_d_;
3.  desensitized (with probability *D*): no current is flowing, no activation is possible, recovery will occur stochastically at rat *Γ*_r_.

**Figure 1.**
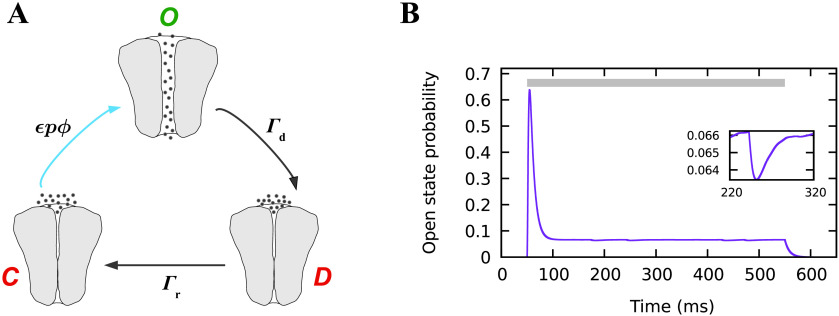
Model of light-sensitive channels. (A) The dynamics of the three-state model for ChR2. The light blue arrow indicates a light-dependent transition. (B) Trace of the open, state probability in response to 5, mW/mm^2^ light stimulation for 500 ms (shown by gray bar). Sustained illumination evokes an initial peak followed by a steady state caused by desensitization. Switching off the light stimulation leads to definite channel closing and a monoexponential decay of the open, state probability back to the baseline. The small fluctuations in the steady state, as magnified in the inset, are caused by the voltage dependence of desensitization.

A sketch of these dynamics is shown in Figure 1. The dynamics can be described mathematically by a reduced two-dimensional system, using the condition that the probabilities *C*, *O*, and *D* add up to one:

$$\frac{dO(t)}{dt}=\epsilon \;p(t)\phi (t)-\left[\epsilon \;p(t)\phi (t)+\Gamma_{\text{d}}(V)\right]\cdot O(t)-\epsilon \;p(t)\phi (t)\cdot D(t),$$ (1)

$$\frac{dD(t)}{dt}=\Gamma_{\text{d}}(V)\cdot O(t)-\Gamma_{\text{r}}\cdot D(t).$$ (2)

A list of the most important parameters used in our simulations is given in Table 1.

**Table 1.** The most important parameters used in our simulations

**Symbol**	**Value**	**Description**
Δ*t*	0.1 ms, 0.01 ms	Duration of one time step for network and single-neuron simulations, respectively
*t*_max_	20 s	Total duration of the simulations
*t*_pulse_	4 ms	Duration of one light pulse
*λ*_max_	470 nm	Wavelength of light, absorption maximum of ChR2/H134R (Williams et al., 2013)
*τ*_ChR2_	1.3 ms	Activation time constant for channels (Nikolic et al., 2009)
*σ*_ret_	12 ⋅ 10^−20^ m^2^	ChR2 retinal absorption cross-section (Williams et al., 2013)
*g*_ChR2_	100 fS	Single channel conductance (Lin, 2011)
*w*_loss_	1.3	Loss factor accounting for the environment of a channel (Nikolic et al., 2009)
Γ_d,0_	126.74 1/s	Desensitization rate at a potential of −70 mV (Tchumatchenko et al., 2013)
Γ_r_	8.38 1/s	Recovery rate (Tchumatchenko et al., 2013)
*ϵ*	0.5	Quantum efficiency for channel opening (Nikolic et al., 2009)
*τ*_m_	10 ms	Membrane time constant
*τ*_ref_	3 ms	Duration of the refractory period
*g*_m_	0.1 μS	Absolute membrane conductance, excluding ChR2 conductances
*V*_rev_	−65 mV	Reversal (equilibrium) potential
*V*_reset_	−70 mV	Reset potential
*V*_th_	−55 mV	Threshold potential to be crossed for spiking
*N*_ChR2_	60 000, 300 000	Number of ChR2 channels within one neuron
*σ*_wn_	0.01 nA	Standard deviation for Gaussian noise in external fluctuations
*I*_0_	0.914576 nA	Mean of the external fluctuations (given value is for a single neuron)
*σ*_light_	8 grid units	Standard deviation of the spatial Gaussian distribution of light
*N*_E_	3 600 = 60 × 60	Number of neurons in the excitatory population, aligned in a square
*N*_I_	900	Number of neurons in the inhibitory population
*p*_c_	0.005–0.015	Connectivity (probability of connection between two neurons) (Braitenberg & Schüz, 1998; Le Duigou et al., 2014)
*τ*_syn_	5 ms	Synaptic time constant, also for external fluctuations

*Note*. Unless stated otherwise, the values were used as stated in this table.

The photon flux *ϕ*(*t*) is related to the light intensity *E*(*t*) that is used for stimulation:

$$\phi (t)=\frac{\sigma_{\text{ret}}\cdot \lambda_{\text{max}}}{h\cdot c\cdot w_{\text{loss}}}\cdot E(t).$$ (3)

Hence, the light intensity reaching the ChR2 receptor is a product between the experimentally applied light intensity, the scattering loss factor *w*_loss_, and the ChR2 absorption cross-section *σ*_ret_. The two latter factors determine the fraction of the light intensity that can be absorbed by the ChR2 molecules. The constants *h* = 6.62606957 ⋅ 10^−34^ Js and *c* = 299 792 458 m/s are the Planck constant and the speed of light in vacuum, respectively.

Effectively, only a fraction *ϵ* of the quanta absorbed by the light receptor of a channel contributes to channel opening. By an activation function *p*(*t*), the model can account for noninstantaneous adaptation to light, which is of special importance when short light pulses are used (Nikolic et al., 2009):

$$p(t)=1-\exp\left(-\frac{t-t_{\text{light}}}{\tau_{\text{ChR2}}}\right).$$ (4)

Furthermore, it can account for voltage-dependent deactivation (Mattis et al., 2011) through a voltage dependence of the desensitization rate Γ_d_ (Tchumatchenko et al., 2013). The voltage dependence of the desensitization rate is given by:

$$\Gamma_{\text{d}}(V)=\Gamma_{\text{d,0}}\left(1-0.0056\;{\text{mV}}^{-1}\left(V+70\;\text{mV}\right)\right).$$ (5)

Light-induced channel opening gives rise to a depolarizing photocurrent (Nikolic et al., 2009):

$$I_{\text{ChR2}}(t,V)=-(V-E_{\text{ChR2}})\cdot N_{\text{ChR2}}\cdot g_{\text{ChR2}}\cdot O(t).$$ (6)

The photocurrent is proportional to the expression level *N*_ChR2_, which is the total number of channels in the neuron. The product of *N*_ChR2_with the probability of a channel to be in the open state, *O*(*t*), represents the number of channels in that state. In addition, the photocurrent recursively depends on the membrane potential *V* and on the conductance *g*_ChR2_ of a single channel. Via the photocurrent, the stimulus imposes its temporal structure on the membrane current of the neuron and consequently on its activity trace. The current enters the Leaky integrate-and-fire neuron, which has a membrane potential described by the following equation:

$$C_{\text{m}}\frac{dV(t)}{dt}=-g_{\text{m}}(V(t)-V_{\text{rev}})+I_{\text{syn}}(t)+I_{\text{ChR2}}(t,V).$$ (7)

The membrane potential further depends on the membrane capacitance *C*_m_, the membrane conductance *g*_m_, the reversal potential *V*_rev_, and the current *I*_syn_(*t*) entering through synapses. Parameter values are given in Table 1.

To account for synaptic inputs, a current *I*_syn_(*t*) enters the Leaky integrate-and-fire equation. This current can comprise different contributions. We modeled external contributions from outside the simulated network as an Ornstein–Uhlenbeck process in the following way:

$$\tau_{\text{syn}}\frac{dI_{\text{ext}}(t)}{dt}=I_{0}-I_{\text{ext}}(t)+\sigma_{\text{wn}}\cdot \Gamma (t).$$ (8)

The Ornstein–Uhlenbeck statistics (colored noise) are suitable for this purpose because its power spectrum has been shown to be consistent with experimental recordings of cortical neurons (Destexhe, Rudolph, & Paré, 2003). The Ornstein–Uhlenbeck process contains the synaptic time constant *τ*_syn_, a mean current *I*_0_, and Gaussian white noise Γ(*t*) with standard deviation *σ*_wn_and mean 0.

The external input causes spiking dynamics to occur even in the absence of light stimulation. We adjusted the mean current *I*_0_ for all simulations, such that unstimulated neurons were firing at a mean rate of 5 Hz. For single neurons that are not simulated in a network, the synaptic input equals the external input: *I*_syn_(*t*) = *I*_ext_(*t*).

**Figure 2.**
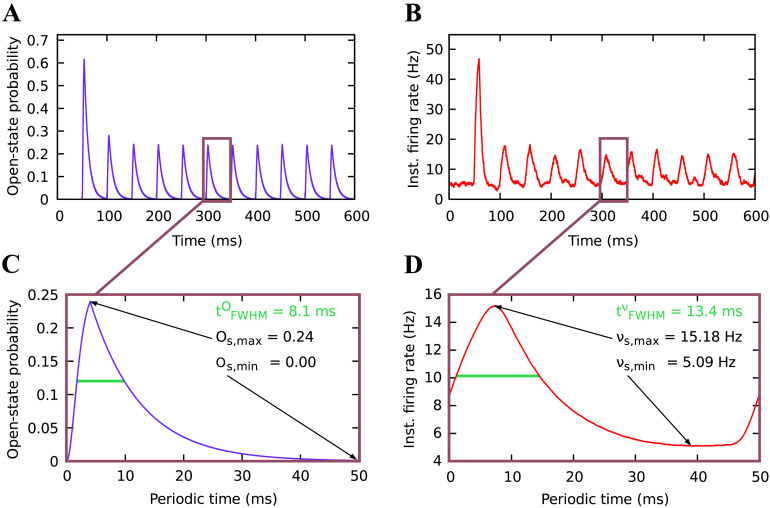
Characteristics of the response dynamics of ChR2 channels and neurons after the steady state has been reached. (A) Open-state probability of ChR2 channels and (B) activity of the related neuron, following stimulation with a frequency of 20 Hz and a light intensity of 5 mW/mm^2^ (averaged over 900 trials). A steady state is reached quickly after the onset dynamics. (C) Course of a periodic pulse in the steady-state open-state probability of the channels, extracted from the temporal course of a simulation as shown in A. Response pulse duration (full width at half maximum), maximum, and minimum value of the steady-state open-state probability can be determined from this diagram, as indicated by the arrows and the green bar. (D) Course of a periodic pulse in the steady-state activity of a single neuron, averaged over all pulses within 20 s from the temporal course of a simulation as shown in B. Pulse duration, maximum, and minimum of the steady-state activity can be determined from this diagram.

In order to characterize the long-term activity dynamics of a single neuron, we examined three measures of the neuronal activity in the steady state. These are the response minimum, which is the lowest value the steady-state firing rate takes under persistent stimulation, the response maximum, which is the total height of the firing rate response pulses evoked by stimulus pulses, and the duration of those pulses, measured by the full width at half maximum. We averaged the pulses across the whole steady state and over 900 trials. To draw a comparison between the impact of the neuron dynamics and the channel dynamics, we determined the same three measures from the pulses in the steady-state open-state probability of a channel. Examples of the pulses in the steady-state open-state probability and the steady-state activity are shown in Figure 2C, D.

The onset phase of the light stimulation causes a strong but very brief spiking response lasting approximately 100 ms (see Figure 2A and B). This onset response vanishes quickly and its features are different from the steady-state response that follows. Therefore, to measure the experimentally more representative long-term spiking response, we considered the spikes which followed the onset phase and imposed a wait time in our simulations of approximately 100 ms (see Figure 2).

We used stimulation protocols consisting of temporally rectangular light pulses, delivered with different frequencies and light intensities. The duration of the pulses was kept constant at 4 ms, which is a value that has been used in experiments when employing moderately short light pulses (Boyden et al., 2005; Gunaydin et al., 2010). We used a sliding window/boxcar kernel approach to compute the neuronal activity from spike trains (Dayan & Abbott, 2001). This method is particularly useful because it prevents the resulting activity from being dependent on the placement of the time window, as would be the case with peristimulus time histogram spike densities.

### Network Model

**Figure 3.**
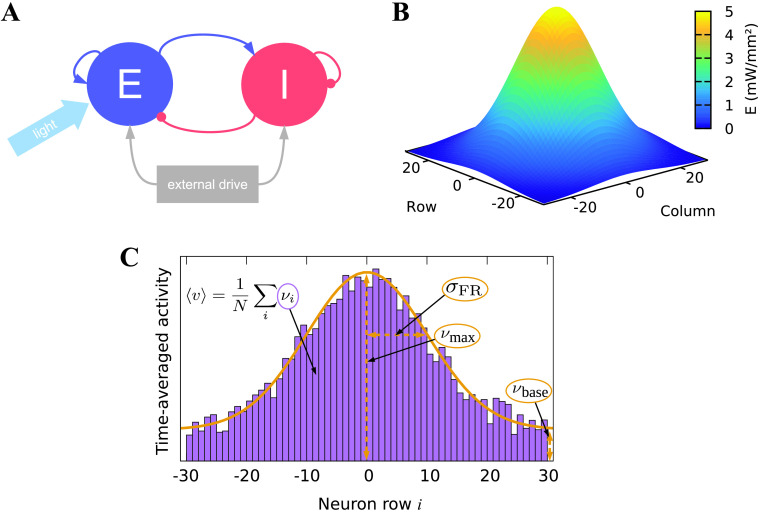
Characteristics of the computational setting. (A) Schematic of the network architecture. The excitatory population (‘E’) is stimulated by light. It is bidirectionally coupled to the inhibitory population (‘I’). Both populations are recurrently coupled and receive external colored noise input, whose power spectrum matches that of neuronal populations (Destexhe et al., 2003). (B) Three-dimensional plot of the Gaussian distribution of light intensity that is used to stimulate the excitatory population of the network (standard deviation of 12 grid units). The number of grid units corresponds to the number of neurons along the axes. In this example, the intensity amplitude is 5 mW/mm^2^. (C) Two-dimensional schematic showing the computation of the population activity 〈*ν*〉, the spatial width *σ*_FR_, the height *ν*_max_, and the baseline *ν*_base_ of the activity distribution. The *N* bar-shaped areas have heights proportional to the mean activity of the respective neuron *i*. The Gaussian curve is fitted to these mean activities. In our simulations, we employed this concept in three dimensions with *N* × *N* neurons.

Our network was represented by a square grid of excitatory neurons, and a population of inhibitory neurons. All neurons were coupled via random recurrent connections (Figure 3A). The probability of a connection *p*_c_ (i.e., the connectivity) was the same for all types of connections (E → E, E → I, I → E, I → I) and was varied across simulations.

As mentioned previously, the neurons receive input accounting for projections from outside the network, modeled by an Ornstein–Uhlenbeck process (Equation 8). In our network simulations, the synaptic current *I*_syn_(*t*) further contains contributions from synapses within the network: *I*_syn_(*t*) = *I*_ext_(*t*) + ∑_*i*_ *I*_int,*i*_(*t*).

We modeled the synaptic contributions by exponential postsynaptic potentials:

$$I_{\text{int},i}(t)=\overset{N}{\underset{j=1}{\sum }}w_{ij}\overset{N_{j}}{\underset{n_{j}=1}{\sum }}\frac{J_{ij}}{\tau_{\text{syn}}}\exp\left(-\frac{t-t_{n_{j}}}{\tau_{\text{syn}}}\right)\Theta (t-t_{n_{j}}).$$ (9)

In this equation, *N* is the number of synapses projecting to neuron *i*, *w*_*ij*_ ∈ 0, 1 is a binary variable specifying the existence of the connection *j* → *i*, *N*_*j*_ is the number of spikes that have occurred in neuron *j*, *J*_*ij*_ is the synaptic coupling strength between neuron *j* and neuron *i* (determined depending on the type of the neurons, as shown in Table 2), and *τ*_syn_ is the synaptic time constant. The coupling strength *J*_*ij*_ is divided by *τ*_syn_ to ensure that for the integration over the whole postsynaptic current, the charge deposited in the postsynaptic neuron is equal to *J*_*ij*_ (Gerstner & Kistler, 2002). The Heaviside theta function Θ(.) accounts for the time of spike occurrence; before the time *t*_*n*_*j*__ at which the *n*_*j*_-th spike of neuron *j* occurs, the term is zero.

**Table 2.** The four different coupling strengths used within the network

***J*_*ij*_/pC**	**From excitatory population (*i* ≡ E)**	**From inhibitory population (*i* ≡ I)**
**To excitatory population (*j* ≡ E)**	0.110	−0.340
**To inhibitory population (*j* ≡ I)**	0.190	−0.540

For stimulation, we again used temporally rectangular light pulses. The intensity of the pulses was spatially modulated through a Gaussian distribution with its maximum in the center of the grid and a standard deviation *σ*_light_, measured in units of the grid index. The distribution is given by (see also Figure 3B):

$$E(r)=Ê\cdot \exp\left(-\frac{r^{2}}{2\cdot \sigma_{\text{light}}^{2}}\right),$$ (10)

where *r* = sgn(*x* − *x*_c_) ⋅ $\sqrt{{\left(x-x_{\text{c}}\right)}^{2}+{\left(y-y_{\text{c}}\right)}^{2}}$ describes the distance of a neuron to the center of the Gaussian light stimulus at (*x*_c_|*y*_c_); the signum function sgn(*x* − *x*_c_) is employed for visualization purposes (cf. plots in Results section). We varied the amplitude (i.e., the maximum light intensity) *Ê* of the Gaussian distribution across simulations. The stimulation frequency was maintained at 50 Hz, which is a value commonly used in experiments for excitatory optogenetic stimulation (Lin et al., 2009; Zhang et al., 2006).

Complementary to the light stimulation, we used a Gaussian fit function to obtain the width, height, and baseline of the spatial activity distribution:

$$\nu (r)=\left(\nu_{\max}-\nu_{\text{base}}\right)\cdot \exp\left(-\frac{r^{2}}{2\cdot \sigma_{\text{FR}}^{2}}\right)+\nu_{\text{base}},$$ (11)

where *σ*_FR_, *ν*_max_ and *ν*_base_ are fit parameters. We estimated a tolerance of 0.2 Hz for the activity data. The computation of the width and the height of the activity distribution and of the population activity is visualized in a two-dimensional sketch in Figure 3C. An example of a light stimulus used for the calculations is displayed in Figure 3B.

## RESULTS

### Response Characteristics of a Single Neuron

First, we investigated how a single neuron containing a given number of ChR2 channels responds to the application of pulsed light stimuli. We used the Leaky integrate-and-fire model to describe the neuron. We selected this neuron model because it reproduces various properties of neocortical pyramidal cells (Gerstner & Naud, 2009; Tchumatchenko et al., 2011) and because of its outstanding numerical speed in network simulations. For the ChR2 channels, we used a probabilistic three-state Markov model, based on previous studies (Nikolic et al., 2009; Tchumatchenko et al., 2013).

**Figure 4.**
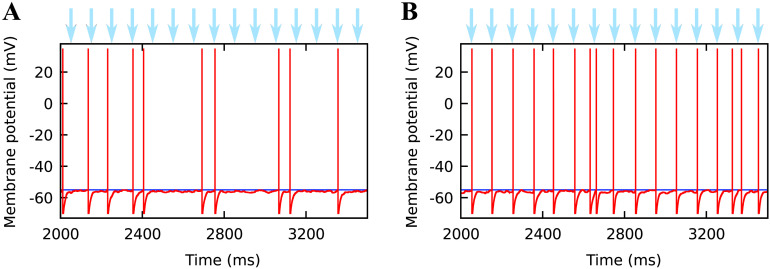
Relationship between light stimulation (light pulses of intensity 2 mW/mm^2^ at 10 Hz, indicated by light blue arrows) and the membrane potential (red traces) of a single neuron. The threshold potential is indicated by the blue line. The plot starts from 2,000 ms to be sure to show pure steady-state dynamics. (A) For 60,000 channels per neuron, the depolarization caused by light is not sufficient to evoke spiking. Nonetheless, random spikes occur due to external input. (B) For 300,000 channels per neuron, the depolarization caused by light is sufficient to evoke spiking, which leads to synchronization of light stimulation and firing.

As a first test for our model, we evaluated the impact of the expression level on the membrane potential dynamics. The results showed that at a low expression level of 60,000 channels, the number of channels was not sufficient to depolarize the neuron strongly enough to make it fire in synchrony with the frequency of light stimulation. At a higher expression level of 300,000 channels, synchronized firing occurred. Membrane potential traces for these two cases are shown in Figure 4.

Under sustained stimulation with a certain frequency and intensity, the dynamics of the open-state probability as well as the firing rate dynamics reached a steady state. In this steady state, we characterized the activity response by measuring the minimum firing rate, the pulse duration, and the maximum of the firing rate pulses in response to light pulses (cf. Figure 1B, Methods). As we varied the frequency and intensity of the light pulses, we recorded the response characteristics for a broad range of stimulus protocols.

**Figure 5.**
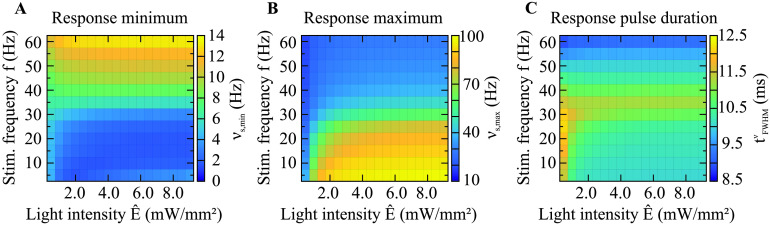
The nonlinear dependence of the steady-state firing rate response of a neuron holding 300,000 ChR2 channels on stimulus frequency and light intensity. The data points were obtained by averaging over 900 neurons and over the response cycles within 20 s after stimulus onset (e.g., 400 cycles at 20-Hz stimulation), following the method visualized in Figure 2D. (A) The activity response minimum increases with increasing frequency. Light intensity does not have a major impact on the response minimum. (B) Remarkably, as the frequency increases, the activity response maximum decreases. The response maximum grows monotonically with the light intensity until it saturates. (C) The response pulse duration increases with frequency at low frequencies, reaches a maximum and then decreases with frequency at high frequencies. Thus, the relation to frequency is nonmonotonic. Regarding increasing light intensity, there is a trend that the pulse duration decreases until saturation.

We found that the activity response of a single neuron under optogenetic excitation exhibited characteristics that might be considered surprising or counterintuitive. In a pulsed stimulation protocol, the minimum of the evoked spiking activity *ν*_s,min_ increased with higher stimulation frequency (Figure 5A), while the maximum of the evoked activity pulses *ν*_s,max_ decreased (Figure 5B). Furthermore, it is remarkable that the duration of the firing rate pulses $t_{\text{FWHM}}^{\nu }$ exhibited a nonmonotonic relation in response to varying stimulation frequency (Figure 5C). In Figure 5 we show results for neurons with 300,000 channels while we found qualitatively similar behavior also for 60,000 channels per neuron.

**Figure 6.**
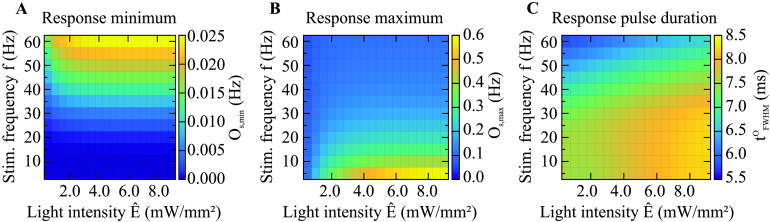
The nonlinear dependence of the steady-state open-state dynamics of the ChR2 channel on stimulus frequency and light intensity. The method for obtaining the open-state statistics is visualized in Figure 2C. (A) The response minimum of the open-state probability increases with increasing frequency. Light intensity causes a slight increase at higher frequencies, but generally only exhibits a minor effect on the response minimum. (B) The response maximum decreases as the frequency increases. It scales monotonically with light intensity until it saturates. (C) The response pulse duration decreases with frequency, and increases with light intensity until saturation.

One could expect that the activity response increases its minimum with increasing stimulation frequency, since a higher frequency should lead to more depolarization because it raises the level of continuous activity (Figure 5A). This is also what we found. Considering the dependence on the light intensity, there is a slight decrease in the activity response minimum for lower light intensity. The response minimum of the open-state probability showed similar behavior (Figure 6A).

The detailed behavior of the response maxima, however, is more intriguing. Our simulations show that as the stimulation frequency increases, the absolute height of the response pulses (i.e., the response maximum) decreases as it approaches the response minimum (Figure 5B). For very high stimulation frequencies, the response maximum and minimum have to be equal because above a certain frequency, which depends on the light pulse duration (250 Hz for 4 ms), pulsed stimulation would become in fact constant and the response pulses would vanish as well. Studying the dependence on the light intensity, we found a monotonic increase of the response maximum, leading into saturation. The response maximum of the open-state probability showed similar behavior (Figure 6B).

Studying the activity pulse duration (Figure 5C), we first found that it was longer than the duration of the stimulus pulses, which had a constant duration of 4 ms. In addition, we made a surprising discovery. The relationship of the activity pulse duration to the frequency was not monotonic, unlike the monotonic increase of the response minimum and decrease of the response maximum with increasing frequency. Instead, the pulse duration exhibited a maximum whose width and height depend on the stimulation frequency and light intensity. The comparison with the pulse duration of the open-state probability (Figure 6C), which does not exhibit such a maximum, suggests that the occurrence of the maximum is due to the restricted firing capability of neurons.

We find that for increasing light intensities, the firing rate response and the open-state probability tend to approach a constant value. Hence, it seems that the occupancy of the open state saturates (Figure 6). This can be explained by the limited number of channels that are in the closed state, from which they transition to the open state. In the regime of high light intensities, the probability of the desensitized state is much larger than that of the closed state because of the high opening and desensitization rates on the one hand and the slow recovery from the desensitized to the closed state on the other hand. We can understand this effect by evaluating the time-averaged opening rate:

$$\langle \Gamma_{\text{o}}\rangle :=\frac{1}{T}\overset{T}{\underset{0}{\int }}\epsilon p(t)\phi (t)dt=\epsilon \hat{\phi }\cdot f\left(t_{\text{pulse}}+\tau_{\text{ChR2}}\cdot \left(e^{-t_{\text{pulse}}/\tau_{\text{ChR2}}}-1\right)\right),$$ (12)

where $\hat{\phi }$ is the photon flux during the stimulus pulses of duration *t*_pulse_, computed from the light intensity as per equation 3. The duration of the stimulus periods is given by *T* = 1/*f*. Table 3 shows opening rates for different light intensities and stimulus frequencies, which compete with the desensitization rate Γ_d_ ≈ 126.74 1/s and the recovery rate Γ_r_ = 8.38 1/s. To summarize, we find that at higher light intensities, the open state probability of the channels saturates, which is due to the low recovery rate of channels as compared to the opening and desensitization rates.

**Table 3.** Time-averaged opening rate 〈Γ_o_〉 (cf. Equation 12) for different light intensities and stimulus frequencies

***Ê*/mW/mm^2^**	4.0			6.0			8.0		
***f*/Hz**	5	30	60	5	30	60	5	30	60
**〈Γ_o_〉/1/s**	6.03	36.17	72.33	9.04	54.25	108.5	12.06	72.33	144.66

*Note*. Note that 〈Γ_o_〉 ∝ *Ê*, *f*.

The comparison of the steady-state dynamics of the neuronal firing rate (Figure 5) and the open-state probability of a ChR2 channel (Figure 6) shows that the behavior of the response minimum and maximum of the activity seems to be determined predominantly by the channel dynamics. Since the pulses in the firing rate as well as in the open-state probability are longer than the stimulus pulses, some filtering has to occur already at the channel level. Nevertheless, the pulse duration of the firing rate shows nonmonotonic behavior in relation to the stimulus frequency, while the pulse duration of the open-state probability has a monotonic relationship to the stimulus frequency. Hence, the pulses of the firing rate reflect nonlinear neuronal processing. In fact, the pulse duration of the firing rate is smoothed out by the processes that occur in Leaky integrate-and-fire neurons (cf. Equations 6 and 7).

### Spatial Extent of the Activity Response of a Network

**Figure 7.**
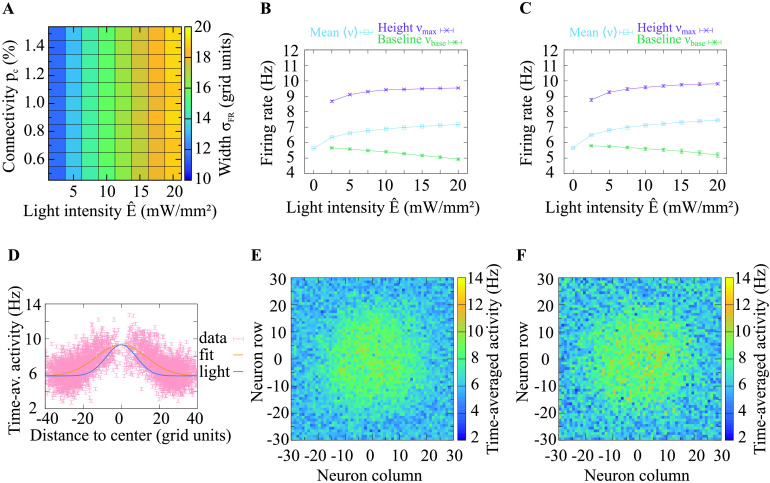
Network response if neurons express on average 60,000 ChR2 channels per neuron. The firing rate distribution is broader than the light distribution but narrower than the response at a higher expression level (Figure 8). (A) The width *σ*_FR_ of the spatial distribution of activities (cf. Figure 3C) is much larger than the width of the light stimulation. This suggests that neuronal activity spreads widely following narrow light stimulation. The connection probability *p*_c_ has almost no impact on the response width *σ*_FR_. The width rises as the light intensity *E* increases. (B) Height *ν*_max_ and baseline *ν*_base_ of the spatial distribution of activities, and population activity 〈*ν*〉, depicted across different light intensities; *p*_c_ = 0.5%. (C) Height and baseline of the spatial activity distribution and the population activity are shown across different light intensities; *p*_c_ = 1.0%. (D) Gaussian fit to the spatial distribution of activities resulting from *p*_c_ = 1.0% and *Ê* = 5.0 mW/mm^2^. The data points denote the time-averaged activity of neurons and their distance to the center of the stimulation (in units of the grid index). The width, height, and baseline of the distribution are estimated by the standard deviation, amplitude, and vertical shift of the Gaussian fit, respectively. The light distribution that evoked the activity distribution is shown to enable comparing the widths. This indicates that a narrow stimulus distribution evokes a broad response distribution (here more than 1.5 times as broad). (E, F) Distributions of time-averaged activities for maximum light intensity *Ê* = 5.0 mW/mm^2^ and connectivity *p*_c_ = 0.5% and *p*_c_ = 1.0%, respectively. The data in A, B, and C were averaged over 10 trials. In some cases, the standard deviation is very small, such that the error bars are covered by the lines. The spatial stimulation width was kept constant across figures.

**Figure 8.**
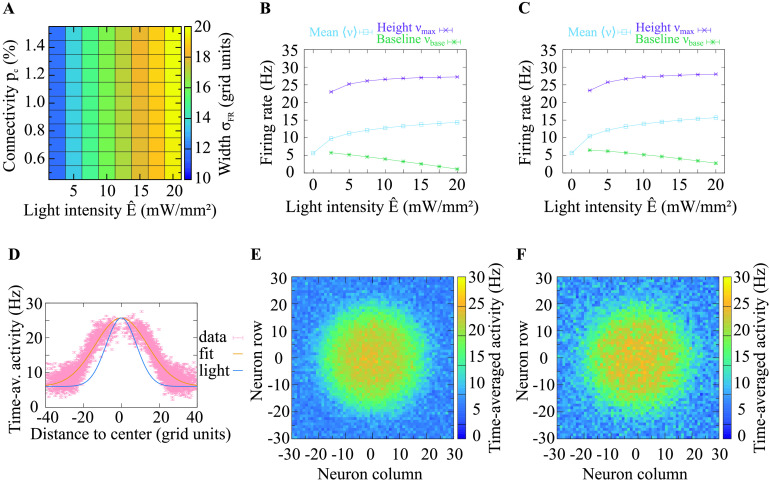
Network response if neurons express on average 300,000 ChR2 channels per neuron. The firing rate distribution is broader than the light distribution and broader than the activity profile we obtained at a lower expression level (Figure 7). (A) The width *σ*_FR_ of the spatial distribution of activities (cf. Figure 3C) is much larger than the width of the light stimulation. This suggests that neuronal activity spreads widely following narrow light stimulation. The response width *σ*_FR_ is almost independent of the connection probability *p*_c_. The width rises as the light intensity *E* increases. (B) Height *ν*_max_ and baseline *ν*_base_ of the spatial distribution of activities, and population activity 〈*ν*〉 depicted across different light intensities; *p*_c_ = 0.5%. (C) Height and baseline of the spatial activity distribution and the population activity are shown across different light intensities; *p*_c_ = 1.0%. (D) Gaussian fit to the spatial distribution of activities resulting from *p*_c_ = 1.0% and *Ê* = 5.0 mW/mm^2^. The data points denote the time-averaged activity of neurons and their distance to the center of the stimulation (in units of the grid index). The width, height, and baseline of the distribution are estimated by the standard deviation, amplitude, and vertical shift of the Gaussian fit, respectively. The light distribution that evoked the activity distribution is shown to enable comparing the widths. This indicates that a narrow stimulus distribution evokes a broad response distribution (here almost 2 times as broad). (E and F) Distributions of time-averaged activities for maximum light intensity *Ê* = 5.0 mW/mm^2^ and connectivity *p*_c_ = 0.5% and *p*_c_ = 1.0%, respectively. The data in A, B, and C were averaged over ten trials. In some cases, the standard deviation is very small, such that the error bars are covered by the lines. The spatial stimulation width was kept constant across figures.

At the network level, we investigated the spatial distribution of activity following excitation through a spatially Gaussian-distributed light stimulus. As for the investigation of a single neuron, we used pulsed light stimuli at different light intensities, but now maintained their frequency at 50 Hz. Additionally, we spatially modulated the intensity with a Gaussian distribution. The center of the Gaussian spatial distribution conformed with the center of the square grid of excitatory neurons. We observed the resulting activity levels of the excitatory neurons following this stimulation paradigm. By averaging over the time-averaged activities of all individual neurons, we obtained the population activity 〈*ν*〉. To further analyze the network response, we sought a measure for the spatial distribution of evoked activity. To this end, we fitted different functions to the evoked activity and compared the *R*^2^ values of these functional fits. We found that the Gaussian had the highest *R*^2^ values (sample values for *p*_c_ = 1%, *Ê* = 5 mW/mm^2^, and 60,000 channels: Gaussian 0.373, Lorentzian 0.363, Logistic distribution 0.368). Thus, we used a Gaussian fit function to describe the spatial distribution of the evoked activity (see Equation 11). We used the standard deviation *σ*_FR_ of this fit function to measure the width, the amplitude *ν*_max_ to measure the maximum, and the vertical shift *ν*_base_ to measure the baseline of the activity distribution. Light distributions, resulting activity distributions and Gaussian fit functions to the activity distributions are shown in Figures 7D and Figure 8D. These figures provide a glimpse on our main finding that the spatial width of the evoked activity distribution is considerably larger than the width of the light stimulus.

We found that the population activity *ν*〉 exhibited the same monotonically increasing behavior as a function of the maximum light intensity for both expression levels (Figures 7B, C and 8B, C). Increasing the connection probability *p*_c_ affected the magnitude but not the shape of the activity profile. We obtained a similar response for the height of the spatial activity distribution (*ν*_max_). The baseline in the activity distributions (*ν*_*base*_) declined as the peak light intensity increased, which could be explained by increased inhibition in the network evoked by the increased excitatory population activity. The width of the spatial activity distribution (*σ*_FR_) increased monotonically with growing light intensity (Figures 7A and 8A). Varying the connection probability had a weak effect on the peak amplitude of the response, but not the shape of the response function. In the considered parameter space, the response width was between 1.5 and 2.5 times the width of the light stimulus (*σ*_light_ = 8 grid units). To summarize, our results revealed that for a large set of parameters, light stimulation evokes substantially broader firing activity responses.

## DISCUSSION

Optogenetic manipulation of neural network activity has become a widely used method to modulate neuronal activity in vivo (Masseck, 2018). One of the most commonly employed optogenetic tools is still channelrhodopsin-2 (ChR2), a blue-light-gated cation-selective ion channel from a species of green algae (*Chlamydomonas reinhardtii*) which conducts H^+^, Na^+^, K^+^, and Ca^2+^ (Nagel et al., 2003). Historically, ChR2 was the first optogenetic channel used in neurons, and it has become the basis for other ChR variants (Masseck, 2018). Anticipating the effectiveness of an optogenetic stimulation and interpreting its outcome in vivo is often difficult because of the influences of channel activation, the single-neuron excitability, and the recurrent network dynamics. These effects interact with one another and can lead to a complex set of outcomes. Analyzing the stimulus-response relationship of neurons is of outstanding importance to theoreticians who aim to model and understand network dynamics following optogenetic stimulation, as well as for experimentalists who wish to alter neuronal activity in brain tissue in a desired way. Here, we present a spiking model and its analysis at the network level in order to help predict the outcome of excitatory optogenetic manipulation. Our computational model considers recurrent networks of excitatory and inhibitory neurons, in which excitatory neurons express light-sensitive ChR2/H134R channels.

To accurately represent the physiological properties of light-stimulated pyramidal neurons, we chose the Leaky integrate-and-fire (LIF) model. This choice is due to the observation that LIF neurons can capture the broad range of frequencies that are reliably encoded by cortical pyramidal neurons (0–200 Hz frequency range; Tchumatchenko et al., 2011), while other more detailed, conductance-based models, including the Hodgkin–Huxley models, fail to represent the dynamical response of cortical neurons (Fourcaud-Trocmé et al., 2003). Furthermore, the integrate-and-fire type models have been shown to accurately reproduce the experimentally recorded spike pattern (Gerstner & Naud, 2009) and thereby offer various computational advantages compared to more detailed conductance-based models. To model synapses, we used a monoexponential model, introducing postsynaptic currents with finite decay time. Because this model facilitates discretization in numerical computations, it is much faster than slightly more detailed alpha-function-based models. The LIF model parameters which we used correspond to the irregular asynchronous regime of cortical neurons: synaptic strength and time constant are chosen so that they account for the amplitude and width of postsynaptic potentials of AMPA, NMDA, and GABA synaptic currents (Gerstner & Kistler, 2002; London, Roth, Beeren, Häusser, & Latham, 2010; Roth & van Rossum, 2009). Moreover, the physiological parameters such as membrane time constant and targeted coefficient of variation range (0.5–1) were chosen to match experimental reports for the cortex (Stevens & Zador, 1998). The LIF model has, as well as in vivo neurons, two important parameter regimes that shape the spiking response of neurons: the noise-driven and the mean-driven regime (Petersen & Berg, 2016). In our study, neurons operate in the noise-driven regime that is typical for cortical neurons in vivo. Therefore, as long as the parameters stay within this biologically plausible regime, our prior work (Herfurth & Tchumatchenko, 2019; Tchumatchenko & Wolf, 2011) and the work of others (Brunel, Chance, Fourcaud, & Abbott, 2001; Gerstner & Kistler, 2002) suggests that one can expect similar results because the shape of the frequency response function is largely preserved across a broad range of membrane time constants, firing rates, and noise levels. To facilitate the exploration of other spiking models or parameter regimes in combination with optogenetic stimulation, we release our program code, so that our readers can consider the effects of any other custom neuron model on the recurrent network activity and, if necessary, adapt the parameters to any particular value of interest (on instructions see the file CUSTOM_MODELS.txt from the code linked in the Methods section). To model optogenetic light stimulation, we used the three-state model of ChR2 because it is computationally efficient, it describes the monoexponential photocurrent decay of ChR2/H134R (Williams et al., 2013), and it has been shown to reliably reproduce experimentally measured responses to continuously varying stimuli (Tchumatchenko et al., 2013). However, our program code can also be used to implement alternative ChR2 dynamics. Extensions of our model could include a fourth state that can account for biexponential decay of the photocurrent, which is exhibited by some ChR2 variants (Nikolic et al., 2009; Williams et al., 2013), light-assisted recovery, which has been observed for some ChR2 variants (Nagel et al., 2003; Nikolic et al., 2009), or a nonlinear voltage dependence factor to the photocurrent, which could account for specific inward rectification effects (Gradmann, Berndt, Schneider, & Hegemann, 2011; Grossman, Nikolic, Toumazou, & Degenaar, 2011; Lin et al., 2009).

We considered the network activity in response to an optogenetic excitation by spatially Gaussian-distributed light stimulation and studied its spatial profile. We found that the spatial extent, that is, the width, of the network activation can be 1.5 to 2.5 times as large as that of the light source. This indicates that in experiments targeting a particular spatial activation profile the light beam width should be chosen smaller than the intended area of activation, and that unintended co-activation of neighboring regions should be monitored. How small the light beam width will need to be depends on the light intensity and channelrhodopsin expression levels. Interestingly, we found that the response profile and its width depended on the expression levels, while the synaptic connectivity in the stimulated region modulated only the peak height but not the width of the response.

Here, we presented results for a number of basic measures of the activity distribution such as width, height, and baseline, while readers interested in more complex measures such as peak-to-width ratios (*ν*_max_/*σ*_FR_) can use use the program code we provide to explore additional quantities of interest.

To clarify the temporal dynamics of the network response we examined the firing rate dynamics at the single-neuron level. We found that under pulsatile light stimulation, which is commonly used in experiments, the neuronal activity response minimum increases with the stimulation frequency, while the activity response maximum decreases. Our results revealed the existence of local maxima in the stimulus-response relationship, which means that specific stimulus parameters can evoke particularly strong and particularly narrow responses. In particular for the pulse duration of the firing rate response we found that at low stimulation frequencies, the depolarization of neurons is not strong enough to produce sustained activation. This is due to the low-pass-filtering effect of neurons, which are not capable of immediate response to fast, single pulses. Furthermore, we found that the response dynamics saturate with higher light intensities, which is caused by the slow recovery of the channels from desensitization.

Although the number of theoretical studies on optogenetic effects is increasing, no study has yet presented a systematic investigation of the nonlinear effects that are subject to our work. Recently, interesting studies were published which use field-programmable gate array (FPGA) processors to simulate networks of small size with a very detailed neuron model (Luo et al., 2017) or provide a framework which enables simulating optogenetic impact on neurons and networks (Evans, Jarvis, Schultz, & Nikolic, 2016). So far, however, no explicit and quantitative predictions were derived for the optogenetically triggered spatial response of neuronal activity in large networks. Similarly, the effects occurring in the firing rate response of single neurons to light pulses of different frequencies have not been quantified. Providing a model and analysis tools, as well as reporting quantitative predictions that can help in the design and interpretation of optogenetic experiments in recurrent networks and single neurons, was the goal of our study. Since our software package is written in standard C++, it enables very fast computation and will in most cases outperform other tools that are based on higher-level languages such as Python, while offering maximal customizability.

What experimentally relevant effects did we find? To achieve controlled optogenetic stimulation in experiments in vivo, it is helpful to be able to reverse engineer the optogenetic excitation profile from the desired neural activation profile. In our study, we identified three important aspects that are relevant for this procedure. First, the size of the area where the stimulus evokes direct or indirect excitation can be substantially larger than the area covered by light (up to 2.5 times; cf. Figures 7 and 8). For experiments, it is therefore beneficial to start with a small area of excitation and broaden it only when there is evidence that not all intended neurons are reached. Starting with a large light stimulation area bears the risk of unintended co-activation of neighboring areas, since the effective footprint of light on the firing rates of neurons will be much broader than the light profile itself. In addition to the broadening of the spiking activity profile relative to light that we have characterized, further broadening of the area of response is to be expected by light scattering in brain tissue. While this investigation was beyond the scope of this study, it should also be kept in mind when setting up light stimulation protocols. Second, our results indicate that the duration of the firing rate pulses in response to light pulses can be substantially longer than the duration of each light pulse that evokes it. Our analysis has shown that evoking very brief firing rate responses is not possible with the ChR2 variant that we considered in our study (ChR2/H134R). Experiments which rely on millisecond or submillisecond precision of few carefully introduced extra spikes need to either drastically lower the light level such that the integrated firing rate response to light stimulation carries only a few spikes, or choose another optogenetic variant that may allow for a higher precision (Lin, 2011; Masseck, 2018). Third, our results show that the amplitude of the steady-state firing rate response is not only proportional to the light intensity, it is also highly sensitive to the frequency of the light stimulation. Such behavior has also been observed in experiments (Ni et al., 2017). If the experimental aim is to elicit strong excitation pulses in neurons, then it is best to use low stimulation frequencies. Otherwise, if the goal is to elicit maximal numbers of spikes that do not need to occur in pulses, then it is best to use intermediate stimulation frequencies of around 30 Hz, which provide an optimal balance between high response minimum and high response maximum. It is important to know that the response pulses of the spiking response in this stimulation range are long, an observation which needs to be considered when measuring the resonance properties of specific neural populations or investigating the synchronization properties in particular frequency ranges (e.g., Cardin et al., 2009). In summary, our study enables a quantitative prediction of neural network activity that can help guide and interpret the outcome of optogenetic experiments.

## ACKNOWLEDGMENTS

We would like to thank Sara Konrad for advice on network dynamics, Rogier Poorthuis and Hiroshi Ito for scientific discussions, and Leonor Fajardo Rebelo for proofreading help.

## AUTHOR CONTRIBUTIONS

Jannik Luboeinski: Conceptualization; Data curation; Formal analysis; Investigation; Methodology; Software; Validation; Visualization; Writing - Original Draft; Writing - Review & Editing. Tatjana Tchumatchenko: Conceptualization; Formal analysis; Funding acquisition; Methodology; Project administration; Resources; Supervision; Validation; Writing - Original Draft; Writing - Review & Editing.

## FUNDING INFORMATION

Tatjana Tchumatchenko, Max Planck Society. Tatjana Tchumatchenko, German Research foundation via SPP 2041.

## TECHNICAL TERMS

Gaussian light beam: A light beam whose transversal intensity profile follows a Gaussian function.
Optogenetics: Umbrella term for techniques that make use of genetic modifications to introduce light sensitivity into neurons.
Ion channel dynamics: Ion channels undergo transitions between different states, depending on factors like time, membrane voltage or light stimulation.
Channelrhodopsin-2: A molecule complex that naturally occurs in unicellular algae. It can be genetically inserted into neurons and depolarize host neurons upon stimulation with blue light.
Markov model: A Markov model assumes that the transition between two states depend only on the very last state and not on the whole history of states.
Nonlinear function: A mathematical function that cannot be expressed by only using the operations of scalar multiplication and addition.
Light stimulation: Optogenetically modified cells can be excited or inhibited by illuminating them with light of a certain wavelength.
Ornstein–Uhlenbeck process: A stochastic process that has an exponentially decaying autocorrelation function and can therefore be used to model cortical noise.
Population activity: The population activity or population rate is the average firing rate of the neurons in a population.
Neuronal firing rate: The neuronal firing rate is the rate of spikes per time fired by a neuron and can be computed from spike trains, for example, by using a sliding time window.
Monotonically increasing function: A function of a parameter that increases or stays constant, but never decreases, as the parameter increases.
